# Iodine loaded nanoparticles with commercial applicability increase survival in mice cancer models with low degree of side effects

**DOI:** 10.1002/cnr2.1843

**Published:** 2023-06-02

**Authors:** Torkel Falkenberg, Olivia Larsson, Bengt Hedin, Shigeru Shiraki, Takahisa Karita

**Affiliations:** ^1^ Department of Neurobiology, Care Sciences and Society Karolinska Institutet Stockholm Sweden; ^2^ Torfal AB, A Working Lab, Innomedicum Karolinska Institutet Science Park Solna Sweden; ^3^ Adlego Biomedical AB Solna Sweden; ^4^ SDS Life Science Stockholm Sweden; ^5^ Shigeru AG Basel Switzerland

**Keywords:** cytotoxic, drug delivery, molecular iodine, nanoparticles, side effects

## Abstract

**Background:**

The recorded use of iodine in medicine, dates to 5000 BC. Molecular iodine (I_2_) has been claimed to exert an antineoplastic effect that triggers apoptotic and re‐differentiation mechanisms in different types of cancer cells in animal studies. Hitherto, all experiments published have been carried out with I_2_ diluted in water preparations resulting in the administration of ionized iodide, either alone or in combination with low levels of I_2_. To maximize the levels of I_2_ by avoiding water solutions we have managed to develop a colloidal nano particle (NP) loaded with I_2_ with a Z‐average of 7‐23 nm with remarkable stability, preferable osmolality and commercial applicability.

**Aims:**

Here we report the results from formulation and pre‐clinical studies with the rationale: a) to find a tolerable dose of the I_2_ NP system delivered intravenously or per‐orally, and b) to determine if the tolerable doses are efficacious in murine models of cancer.

**Methods and Results:**

A novel drug delivery system with I_2_ NP was formulated and murine cancer models with CT26, MDA‐MB‐231 and LL/2 cells were used to analyse the efficacy. Despite the formulation challenges we were successful in constructing stable NPs loaded with I_2_ which have convincing commercial applicability. We conclude that administration of the NP I_2_ drug delivery system: 1. Blunted tumour growth in a xenograft breast cancer model; 2. Had a significant effect on survival in the orthotopic, syngeneic lung metastasis model; 3. Showed reduced tumour burden in post‐mortem evaluation and; 4. Was associated with low degree of side effects.

**Conclusions:**

Taken all together, our findings indicate that the NP I_2_ drug delivery system may serve as a novel effective cancer treatment with low degree of side effects. This is something which needs further exploration including confirmation in future clinical trials.

## 1 INTRODUCTION

The recorded use of iodine in medicine, dates to 5000 BC when seaweed and sea sponges were used to shrink goitres. Chemically, the term iodine represents any form of the molecule and includes molecular iodine (I_2_), iodide salts (NaI or KI), iodate (NaIO) in addition to lipids or proteins containing iodine such as iodotyrosine or iodolactones. Iodide salts have been studied extensively and are often used in supplementation to entire populations.

Iodine is important in thyroid hormone formation and function in addition to independent physiological effects. Iodine also acts intracellularly as an antioxidant, anti‐inflammatory and proapoptotic agent essential for health and cellular renewal.^1^

Molecular iodine (I_2_) has been claimed to exert an antineoplastic effect that triggers apoptotic and re‐differentiation mechanisms in different types of cancer cells.^2^ Moreover, I_2_ may also serve as an immune modulator. It has been reported that several immune cell types can internalise iodine, which depending on the cellular context, imply antibacterial and anti‐inflammatory effects, or induction of immune response.^3^, ^4^

It is well known that I_2_ supplementation at millimolar concentrations exert beneficial effects in women with mammary fibrocystic disease or mastalgia, without negative effect on their thyroid function or general health. Moreover, it has been demonstrated that such anti‐proliferative and anti‐inflammatory effects can also be seen in models of benign prostatic hyperplasia and cancer in various organs that absorb iodine.^2^ Recent findings show that I_2_ exerts synergistic effects when used as an adjuvant to Doxorubicin antineoplastic treatments in animal models.^5^

Hitherto, all experiments published have been carried out with I_2_ or ions thereof, diluted in water as the only means of formulation and administration per orally. Such iodine solutions mainly contain iodide, such as potassium iodide whereas the ideal formulation would contain predominantly I_2_. Hence, it is questionable if previous published studies with water solutions of iodine have had larger proportions of I_2_.

With this lacuna as a basis, we have managed to develop a colloidal nano particle (NP) loaded with I_2_ with a Z‐average of 7–23 nm with remarkable stability and preferable osmolality with the aim to specifically target and inhibit the growth of cancer cells. There is a high proportion of bound I_2_ and product stability exceeds 6 months of shelf life in room temperature. Manufacturing to the highest standard has been executed by one of Europe's largest commercial manufacturers of extemporaneous medicines.

Here we report the results from formulation and pre‐clinical studies with two main objectives: (a) to find a tolerable dose of the I_2_ NP system delivered intravenously or per‐orally, and (b) to determine if the tolerable doses are efficacious in murine models of cancer.

## 2 MATERIALS AND METHODS

### 2.1 Animals

Female BALB/c, C57BL/6J or athymic nude mice (6–8 weeks of age) were acquired from Charles River (Germany). They were housed in groups of 3 to 8 mice in plastic cages with adsorbent bedding in temperature and light–dark cycle (12:12 h) controlled rooms. Food and water were available ad libitum. Animals were left to acclimatise for at least 5 days before the start of experimental procedures.

Animals were handled in accordance with the Federation for European Laboratory Animal Science Association guidelines. All animal procedures were approved by the local ethics committee at Karolinska Institutet (Stockholms djurförsöksetiska nämnd, ethical permit nos: 17114‐2019 and 7496‐2022).

### 2.2 Test item – Colloidal delivery system formulation

Colloidal NP Iodine (CIP) was formulated by Apotek Produktion & Laboratorier (APL) (Kungens Kurva, Sweden). Briefly, potassium iodine and phospholipids were evaluated as a start of the formulation development work, but when not being successful due to precipitation issues and poor solubility of iodine the evaluation switched to use of the solubilizer Macrogolglycerol Ricinoleate or Kolliphor® EL (KEL) as it is known for its good solubilising properties and the ability to spontaneously form micelles. Different concentrations (10%–30% (w/w)) of KEL were evaluated and the concentration found suitable was 10% (w/w). Different concentrations of iodine, 0.4%–5% (w/w) were evaluated and the suitable concentration was found to be 0.4% (w/w). Formulations containing higher amounts of iodine did separate during storage resulting in a brownish milk‐like preparation or precipitated. Chemicals used for manufacture is described in Table 1.

**TABLE 1:** Chemicals used for CIP manufacture.

Name	Function	Manufacturer	Catalogue number
Iodine	Active ingredient	Merck	1.04760.2500
Macrogolglycerol Ricinoleate	Solubilizer	BASF	30 554 032
Sodium citrate dihydrate	Buffering agent	Jungbunzlauer	100 232/100244
Sodium hydroxide	pH adjuster	Merck	1.37041.1002
Water	Diluent	APL	n.a

Abbreviations: n.a, not applicable.

#### 2.2.1 Manufacturing parameters

The manufacture has been performed according to GLP. The Critical quality attributes (CQA) for the formulation were pH, amount of unbound iodine, total amount iodine, osmolality, micelle size distribution, level of endotoxins and sterility. These parameters were evaluated directly after manufacture for the batches used in the animal studies.

pH was close to physiological values. Total iodine concentration was used to calculate the administered dose of iodine. Osmolality was documented to not be below isotonic values. Micelle size distribution was documented to prove nanoparticle size. Level of endotoxins and sterility were documented to allow non‐harmful microbiological load. pH, osmolality, level of endotoxins and sterility were evaluated according to the European Pharmacopeia. The amount of iodine, unbound and total, was evaluated according to an in‐house validated HPLC method. Micelle size distribution was evaluated according to an in‐house validated dynamic light scattering method.

#### 2.2.2 Manufacturing process for CIP

Macrogolglycerol Ricinoleate was charged in a beaker. The iodine crystals were grinded in a ceramic mortar before adding it to the beaker during continuous stirring with an overhead stirrer. Stirring until dissolved. Water was charged, with or without dissolved buffering salts, in portions to the beaker while stirring continuously. pH was adjusted if necessary. The solution was filtered through a 0.22 micrometer filter and dispended in vials (Table 2).

The suitable formulation named Colloidal NP Iodine Product 1 (CIP1) is a dark orange/brownish solution without visible particles. No change in the micelle size distribution was observed after sterile filtration (data not shown).

**TABLE 2:** Compositions of CIP1, CIP3 and placebo.

CIP1	Amount (mg)
Iodine	4
Macrogolglycerol Ricinoleate	100
Water	To 1 g
**CIP3**	
Iodine	4
Macrogolglycerol Ricinoleate	100
Sodium citrate dihydrate	41.2
Water	To 1 g
**Placebo**	
Macrogolglycerol Ricinoleate	100
Water	To 1 g

The analytical method for determining micell size distribution was dynamic light scattering (DLS), which measures the size of particles suspended in liquid by measuring temporal fluctuations in the intensity of scattered light that reflects the diffusion of the particles. DLS using a Malvern Nano Zetasizer (Malvern Panalytical, United Kingdom) determine the size of particles from 1 nm to 10 μm.

The inherent pH of the formulations is about 2 and the osmolality is hypotonic of around 42 mOsm/kg (data not shown). As seen in Table 3, the micelle size distribution is found to be 7–11 nm (Z‐average) for CIP1 formulations and the corresponding placebos. No effect of pH on micelle size distribution can be seen even when the pH was adjusted using 5 M sodium hydroxide. The micelle size growth of CIP1 was temperature dependent and phase separation occurs after 3 months for samples stored at 25°C/60% RH and after 12 months for samples stored at 5°C/Ambient RH. The amount of total iodine was 0.40%–0.43% (w/w) and the amount of free iodine was 23%–37% (w/w) of the total amount of iodine, meaning that roughly two thirds of iodine is bound to the micelle.

**TABLE 3:** Concentration of iodine (free and total), micelle size distribution and pH for CIP1.

Batch	Concentration iodine (w/w)	D_10_ (nm)	D_50_ (nm)	D_90_ (nm)	Z‐average (nm)	pH
B200304‐01 – Placebo	<0.001	6	8	13	11	6.9
B200305‐01 – Active	0.43 (~37% free)	4	5	9	7	2.1
B200615‐01 – Active	0.405 (~23% free)	—	—	—	—	6.4
B200305‐01 – Active	—	5	7	11	9	6.2
B200820‐01 – Placebo	<0.001	6	9	13	11	6.9
B200820‐02 – Active	0.403 (~27.4% free)	4	5	8	7	2.2

Given the challenges with stability, osmolality and pH, various buffering systems were explored where CIP3 formulated with a citrate buffer to prevent precipitation was successful. The amount of citrate buffer needed was evaluated by titration starting at 100 mM citrate and ending at 140 mM citrate with respect to withstanding change in pH.

The suitable CIP3 formulation evaluated is a dark orange/brownish solution without visible particles. The change in pH after addition of iodine was 0.1 pH unit, and no temperature dependent change was recorded when the samples were stored for the citrate buffer formulation (Table 4). The 140 mM citrate buffer formulation is slightly hypertonic.

**TABLE 4:** Osmolality for CIP3 directly after manufacture, and pH before and after addition of iodine and after storage at 5°C/Ambient RH and 25°C/Ambient RH.

Batch	Osmolality (*n* = 2) T17 (mOsm/kg)	pH T0 (before/after addition of iodine)	pH T17
B220207‐01 Citrate buffer 140 mM 5°C	516	6.52/6.42	6.25
B220207‐01 Citrate buffer 140 mM 25°C	528	6.52/6.42	6.24

**FIGURE 1:**
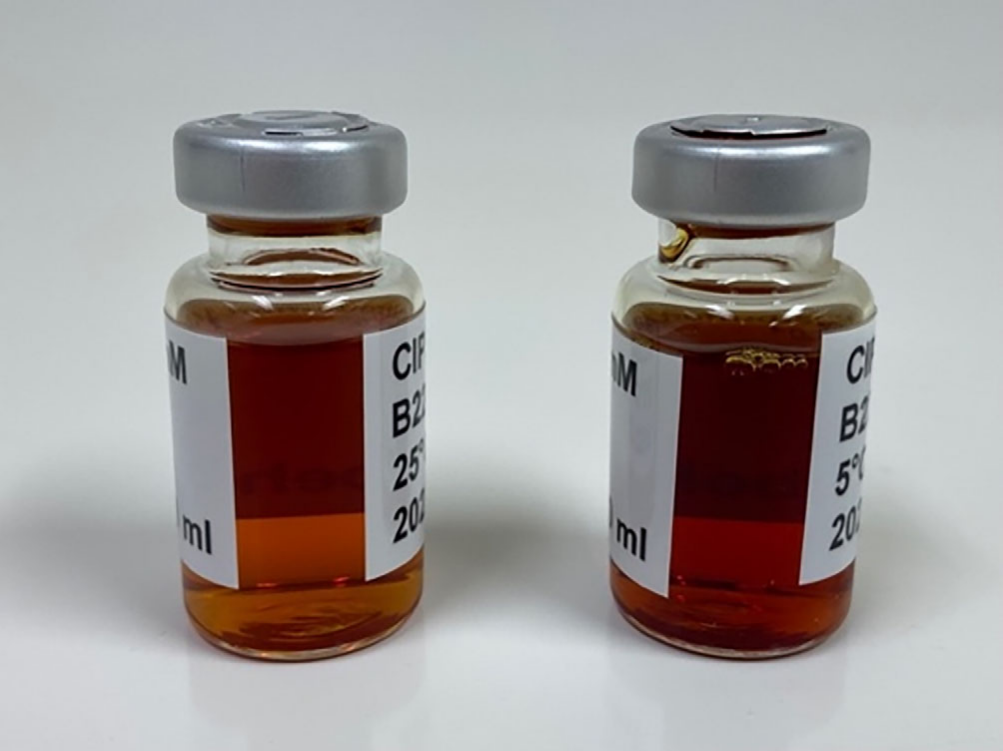
CIP3 – Minor colour difference after 29 weeks between storage at 5°C (right) and 25°C (left).

The micelle size distribution is found to be 15–23 nm (z‐average) for the active CIP3 formulation. No obvious temperature dependent change in micelle size distribution over time, for the samples analysed 4, 10, 29 and 35 weeks after manufacture, was recorded for either active or placebo formulations. In addition, only minor visual changes were observed over the time period (Figure 1). An out of trend result for the data presented after 10 weeks for the active formulation can be seen. This as the micelle size distribution appear to get smaller for the samples stored at 25°C/Ambient RH, but when comparing the micelle distribution for the samples stored at 5°C/Ambient RH a consistent micelle size distribution is seen, and the results at 25°C/Ambient RH after 10 weeks are considered out of expectation (Table 5).

**TABLE 5:** Micelle size distribution of CIP3 and Placebo at different storage conditions and times.

Batch	D_10_ (nm)	D_50_ (nm)	D_90_ (nm)	Z‐average (nm)
B220207‐01 – 10 weeks at 5°C/Ambient RH	8	12	19	17
B220207‐01 – 10 weeks at 25°C/Ambient RH	9	14	24	23
B220207‐01 – 29 weeks at 5°C/Ambient RH	8	11	18	15
B220207‐01 – 29 weeks at 25°C/Ambient RH	8	11	18	15
B220425‐02 – 4 weeks at 25°C/Ambient RH	8	12	21	20
B220425‐02 – 35 weeks at 25°C/Ambient RH	9	13	22	20
B220425‐01 Placebo – 4 weeks at 25°C/Ambient RH	8	12	18	14
B220425‐01 Placebo – 35 weeks at 25°C/ambient RH	8	11	18	17

Patent of CIP is pending, file reference 23DF1309PCT, Patent Cooperation Treaty (PCT) at the Japan Patent Office (JPO).

### 2.3 Experimental procedure

#### 2.3.1 The animal experiments were performed by Adlego Biomedical AB (henceforth referred to as Scantox Solna)

Prior to in vivo administration, CIP1 and CIP3 were diluted to appropriate concentrations. In studies in which CIP1 was pH‐adjusted, 1 M NaOH was used to achieve a pH between 6 and 7. New injectable formulations were made once weekly.

#### 2.3.2 Tolerability

CIP1 was administered via a single or repeated intravenous injection (i.v.) or per‐oral administration by gavage (p.o.), whereafter animals were monitored for changes in body weight and health status at regular intervals. CIP1 was administered at pH 2–3, or adjusted with NaOH to pH 6–7.

#### 2.3.3 Pharmacokinetics

CIP1 was administered i.v. or p.o. Blood samples for isolation of plasma were acquired pre‐administration and at 5 and 30 min, 1, 4, 6, 8 and 24 h after administration. Plasma concentration of iodine was evaluated by ALS Scandinavia (Luleå, Sweden) via inductively coupled sector field plasma mass spectrometry (ICP‐SFMS).

#### 2.3.4 Cancer models

The syngeneic colon cancer model cells (CT26), the xenograft breast cancer model cells (MDA‐MB‐231) and the orthotopic, syngeneic lung metastasis model cells (LL/2) were acquired from American Type Culture Collection (ATCC) (catalogue numbers: CRL‐2638, HTB‐26, CRL‐1642). Cells were cultured in RPMI‐1640 Medium, supplemented with GlutaMAX™ (catalogue number 61870036) 10% foetal bovine serum (FBS; catalogue number: A5256801) and 1% penicillin/streptomycin (P/S; catalogue number: 15140148). MDA‐MB‐231 and LL/2 cells were cultured in DMEM, high glucose (catalogue number: 11965092), supplemented with GlutaMAX™, 10% FBS and 1% P/S. Cells were passaged 2–3 times per week. 3 x 10^5^ CT26 cells were injected subcutaneously in the right rear flank; 1.5 × 10^6^ MDA‐MB‐231 cells were injected into the mammary gland; 3 × 10^5^ LL/2 cells were injected intravenously. All cells were resuspended in PBS (catalogue number: 10010015) prior to injection. Cell media and supplements were obtained from Gibco (ThermoFisher Scientific).

Subcutaneous cancer model: length and width of tumours were evaluated 2 to 3 times per week. Volumes were calculated according to the following formula: L × W × W × 0.44.

Administration of CIP commenced when tumours had reached average volumes of 100 mm^3^ and was performed daily for a period of 14 days. For MDA‐MB‐231 studies, cisplatin, as a positive control, was delivered twice weekly for 3 weeks, intraperitoneally (i.p.) at a dose of 5 mg/kg.

Intravenous model: animals were monitored for changes in health status 2–3 times per week. Treatment commenced 4 days after inoculation of tumour cells and was performed daily for a period of 14 days. For LL/2 studies, cisplatin was delivered twice weekly for 3 weeks, i.p. at a dose of 2 mg/kg.

Three separate experiments were conducted to evaluate the cancerostatic effect of CIP1, delivered per‐orally or intravenously in: (A) a CT26 model; (B) a MDA‐MB‐231 model and; (C) a LL/2 model. The effect of CIP1 and CIP3 were directly compared in a fourth experiment where an LL/2 model was the representative tumour model.

**FIGURE 2:**
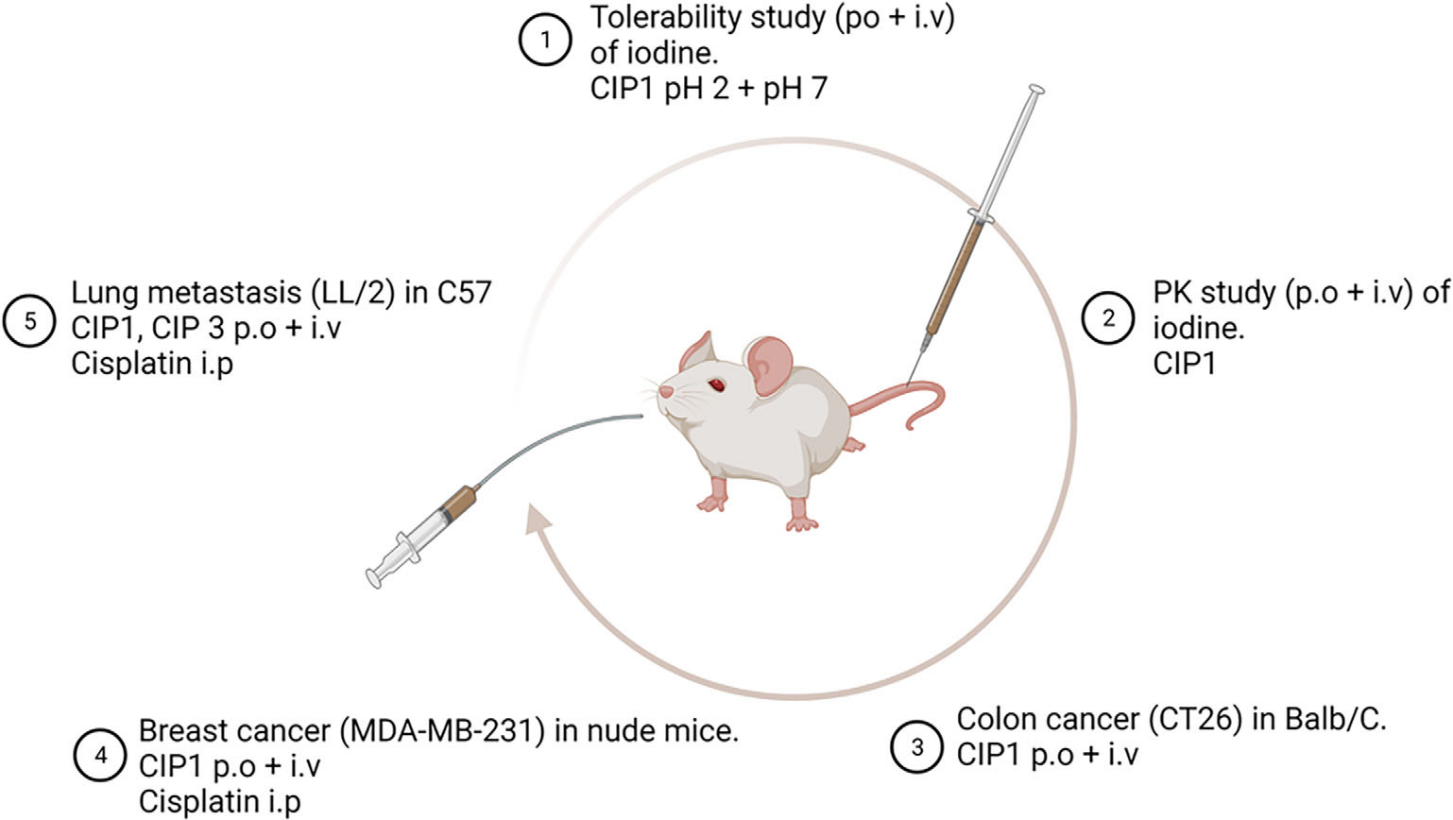
Summary of the animal experiments performed.

All the experiments performed are summarised in Figure 2.

### 2.4 Statistical analysis

Data was analysed using GraphPad Prism software (San Diego, CA). All results are presented as mean ± SEM, unless otherwise specified. For cancer experiments, data was analysed using a two‐way ANOVA, followed by Dunnett's multiple comparison post‐hoc test, to determine if CIP1 or CIP3 was associated with a change in tumour growth rate or pattern of body weight change. A one‐way ANOVA, followed by a Dunnett's multiple comparison post‐hoc test was used to determine whether administration of CIP1 or CIP3 was associated with a change in the number of tumours present in the lung (LL/2 model). Survival was analysed using a Mantel‐Cox (Log‐Rank) test, to determine if CIP1 or CIP3 affect animal survival. A *p*‐value of <.05 was considered significant.

## 3 RESULTS

We have been able to formulate a colloidal NP with I_2_ with appropriate stability allowing for administration of colloidal NP iodine with two main objectives: (a) to find a tolerable dose of CIP, delivered intravenously or per‐orally, and (b) to determine if the tolerable doses are efficacious in murine models of cancer. Initially all experiments were carried out with CIP1 but due to problems of stability and pH adjustments the final experiments included the stable and pH buffered CIP3.

### 3.1 Tolerability

The tolerability of CIP1, given intravenously or per‐orally, at an acidic (2 to 3) or physiological (6 to 7) pH was evaluated. Initial studies assessed the maximum tolerable dose (MTD) following a single intravenous injection of non‐pH adjusted CIP1, through administration of between 0.88 and 85.85 mg/kg. At doses above 2.68 mg/kg, adverse effects, including local swelling and discolouration of the injection site, and a decrease in overall health status, as indicated by presence of piloerection and decreased body weight, were apparent.

Subsequent evaluations assessed the tolerability following repeated dosing of CIP1, as shown in Table 6. CIP1 was administered intravenously or per‐orally daily for up to 14 days. At an acidic pH (pH 2 to 3), doses of 0.88 mg/kg i.v. or 80 mg/kg p.o. were tolerated. At a physiological pH (pH 6 to 7), doses of 4 mg/kg i.v. or 26.7 mg/kg p.o. were tolerated. Higher doses were associated with similar adverse effects as those described above.

**TABLE 6:** Repeated dosing of CIP1.

pH	Route	Minimum dose	Maximum dose	Tolerable dose
2–3	i.v.	0.88	2.90	2.90
2–3	p.o.	8	160	80
6–7	i.v.	4	40	4
6–7	p.o.	26.7	80	26.7

### 3.2 Pharmacokinetics

**FIGURE 3:**
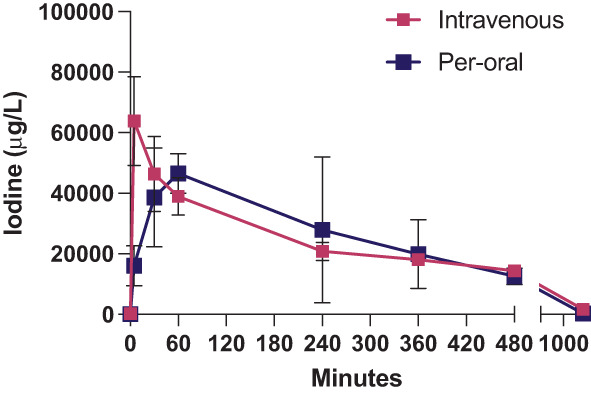
Plasma concentrations of colloidal iodine following intravenous (27 mg/kg) or per‐oral (74.78) administration of CIP1, at an acidic pH.

The pharmacokinetic properties of CIP1 were evaluated following a single intravenous (i.v.) or per‐oral (p.o.) administration (26.7 and 74.8 mg/kg, respectively) (Figure 3). I.v. administration of colloidal iodine was associated with a maximum concentration (C_max_) of 63 600 μg/L, peaking at 0.083 h, and with a half‐life (t_1/2_) of 4.92 h. In comparison, p.o. administration was associated with a C_max_ of 47 900, peaking later at 2.5 h and with a shorter t_1/2_ of 2.34 h. Per milligram of administered colloidal iodine, per‐oral administration was associated with a lower C_max_ as compared to intravenous administration (29 900 vs. 118 000 μg/L/mg). Calculated absolute bioavailability (F) after per‐oral administration was found to be 28.4%.

### 3.3 Cancer models

To determine the cancerostatic potential of CIP1, efficacy was evaluated in three cancer models: a syngeneic colon cancer model (CT26), a xenograft breast cancer model (MDA‐MB‐231) and an orthotopic, syngeneic lung metastasis model (LL/2).

#### 3.3.1 Syngeneic colon cancer model (CT26)

**FIGURE 4:**
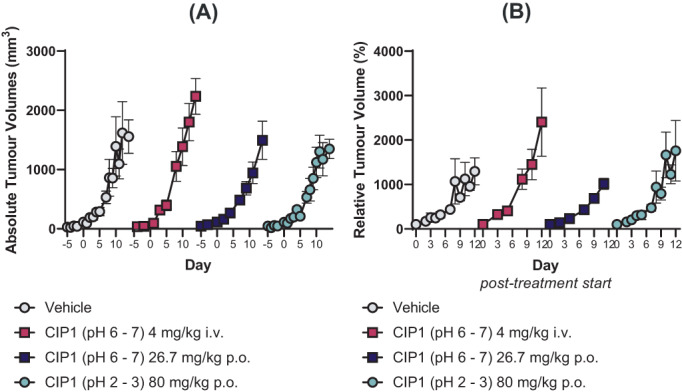
Absolute (A) and relative (B) growth of CT26 tumours following repeated administration of vehicle or CIP1, via intravenous or per‐oral delivery. Relative growth is calculated as percent of volume on Day 0. Data is presented as mean ± SEM, *n* = 9–17.

Per‐oral CIP1, at an acidic pH, and per‐oral and intravenous CIP1, at a physiological pH, was evaluated in a syngeneic model of colon cancer (CT26). In this model, no effect on tumour growth was evident (F_3,270_ = 1.148, *p* = .33) (Figure 4).

#### 3.3.2 Xenograft breast cancer model (MDA‐MB‐231)

As the CT26 model is an aggressive model as such, inability to inhibit CT26 tumour growth is not equivalent to an inability to inhibit cancer growth per se. Therefore, per‐oral and intravenous CIP1, at an acidic and a physiological pH, were evaluated in a slower‐growing xenograft model of breast cancer (MDA‐MB‐231).

**FIGURE 5:**
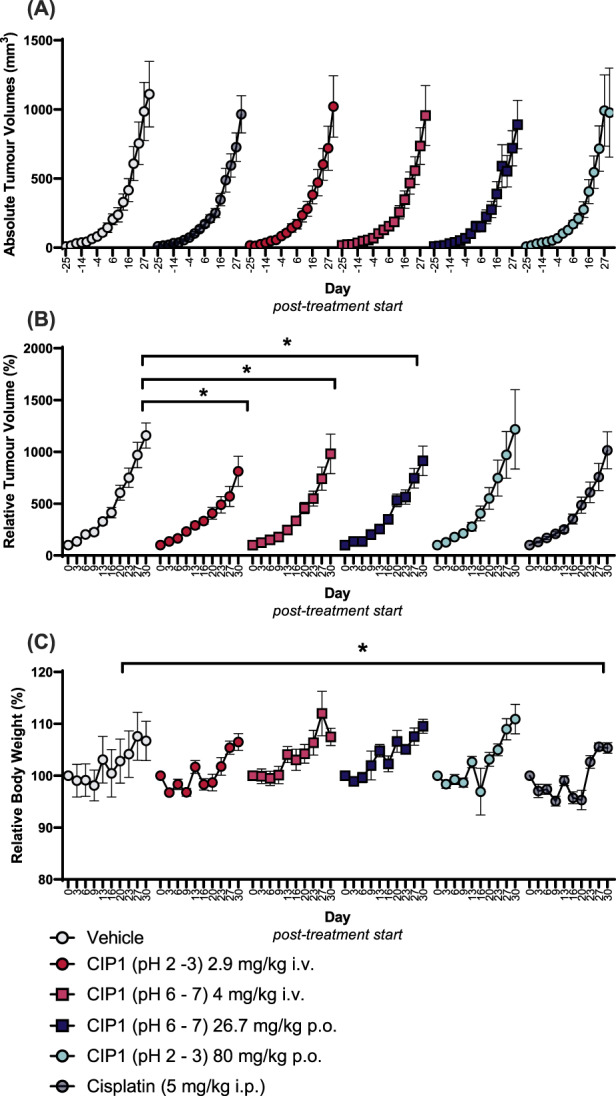
Absolute (A) and relative (B) tumour growth of MDA‐MB‐231 tumours and relative body weight (C) following repeated administration of vehicle or CIP1, via intravenous or per‐oral delivery or cisplatin. Relative growth is calculated as percent of volume/body weight on Day 0. Data is presented as mean ± SEM, *n* = 8. An asterisk indicates a *p*‐value <.05.

Whereas per‐oral CIP1 at pH 2 to 3 had no effect on tumour development, intravenous CIP1, at both physiological (*p* = .024) and acidic pH (*p* = .004), and per‐oral CIP1 at physiological pH (*p* = .049), significantly blunted tumour growth (Figure 5). The most pronounced effect was evident following administration of intravenous CIP1 at an acidic pH. Effects were equivalent to that seen following administration of an established chemotherapeutic agent, cisplatin, but without the adverse health effects, such as decreased body weight (Figure 5C), associated with chemotherapy.

#### 3.3.3 Orthotopic, syngeneic lung metastasis model (LL/2)

**FIGURE 6:**
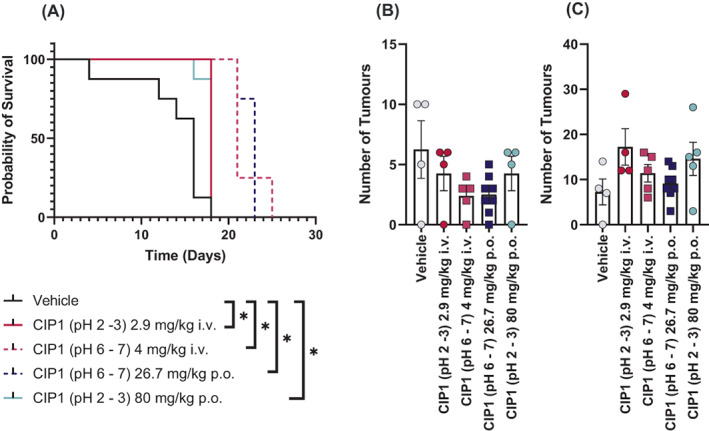
Survival (A) and number of small (B) and large (C) lung tumours, as evaluated post‐mortem, following inoculation with LL/2 cells and repeated administration of vehicle or CIP1, via intravenous or per‐oral delivery. A tumour with a diameter of <3 mm was considered small; a tumour with a diameter of >3 mm was considered large. In bar graphs, data is presented as mean ± SEM, *n* = 8 for survival graph; *n* = 4–8 for tumour burden. An asterisk indicates a *p*‐value <.05.

To further confirm, the effect of CIP1, the cancerostatic properties were evaluated in a syngeneic orthotopic model of lung cancer (LL/2). In a preliminary study, the effect of all tolerable versions of test item were evaluated, including CIP1 at acidic and physiological pH, via per‐oral or intravenous delivery. All test items had a significant effect on survival (Figure 6A); the most prominent effect was seen in animals that were subjected to administration of CIP1 at a physiological pH, which had median survival 21 (i.v.) and 23 (p.o.) days, as compared to a median survival of 16 days for vehicle‐treated animals. However, evaluation of tumour burden in lungs of affected animals showed no significant difference between groups (Figure 6B,C) despite an obvious trend; this may be the result of insufficient group sizes in the evaluation, as animals found dead were not included.

**FIGURE 7:**
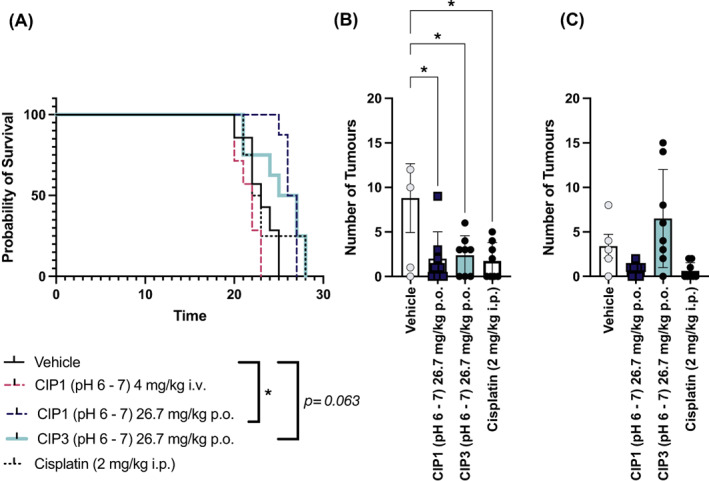
Survival (A) and number of small (B) and large (C) lung tumours, as evaluated post‐mortem, following inoculation with LL/2 cells and repeated administration of vehicle, CIP1 or CIP3, via intravenous or per‐oral delivery, or cisplatin. A tumour with a diameter of <3 mm was considered small; a tumour with a diameter of >3 mm was considered large. In bar graphs, data is presented as mean ± SEM, *n* = 8 for survival graph; *n* = 4–8 for tumour burden. An asterisk indicates a *p*‐value <.05.

A follow‐up study was performed to validate the findings of CIP1 delivered at physiological pH. The second study included a positive control group (cisplatin), as well as a new formulation of CIP (CIP3). CIP3 was formulated in citrate buffer, making it stable at physiological pH, that did not require pH adjustment. As evident in the previous study, administration of CIP1, at physiological pH, significantly affected survival (Figure 6A). The most prominent effect was seen following per‐oral delivery (*p* = .0003), which had median survival of 26.5 days, as compared to a median survival of 23 days for vehicle‐treated animals (Figure 7A). CIP3 showed a similar effect, with a median survival of 26 days, but this did not reach significance (*p* = .063). Cisplatin did not affect survival; however, animals that received cisplatin demonstrated a severe deterioration in body weight (data not shown), which resulted in pre‐term euthanasia, skewing the survival results.

Post‐mortem evaluation of tumour burden further demonstrated a clear effect of CIP1 and CIP3, delivered per‐orally. A significant reduction in presence of small (>3 mm) tumours in the lung was evident for groups that had received per‐oral CIP1 (*p* = .020) and CIP3 (*p* = .028), as well as cisplatin (*p* = .016). Similar effects were evident for CIP1 and cisplatin for large (>3 mm) tumours, but this did not reach significance.

## 4 DISCUSSION

Nanotechnology in health care research and development has resulted in several innovative cancer drug delivery systems such as Doxil™ and Abraxane™ with proven increased drug efficacy, targeting ability and decreasing toxicity.^6^ Some of the promising features for cancer treatment with nanoparticles (NPs) include their ultra‐small size below 100 nm, tumour tissue selectivity and being harmless to coexisting healthy tissue while presenting improvement in their toxicity profile.^7^

For the first time, we report the successful formulation of iodine loaded NPs with a Z‐average of 7–23 nm with remarkable stability and preferable osmolality. Previously, only cyclodextrin particles loaded with iodine with particle shapes of nano cubes and microbars of around 2–4 μm has been reported in the literature for the successful treatment of upper respiratory tract infections.^8^ Our formulation of iodine loaded NPs in the area of cancer open up for large scale production and commercialization provided efficacious effects.

Previous reported cancer experiments with iodine have been restricted to water preparations of iodine despite its low water solubility and the predominant formation of iodine ions in water solutions. It has been suggested that such iodine formulations with predominantly sodium iodide and sodium iodate lead to the generation of molecular iodine in the stomach upon dissolution in the gastric fluid,^9^ something which remains to be proven.

In contrast our unique formulation has a large proportion of I_2_ bound to the NPs. I_2_ is reportedly the active element of iodine, and therefore NPs loaded with iodine has the potential to exert enhanced cancerostatic effects in a drug delivery fashion compared to previous positive trials with water‐based formulations of iodine.^2^ Another advantage is that several studies^10^, ^11^ have shown I_2_ to be less thyrotoxic compared to water soluble iodide which has a different tissue distribution in mammals.

### 4.1 Tolerability and pharmacokinetics

Intravenous administration of CIP1, at an acidic pH, was tolerable at 2.9 mg/kg. At higher doses, animals presented with local adverse reactions, including development of a swollen, blueish black tail, indicative of inflammation and necrosis. This was likely a reaction to the low pH of the test item, which would have likely negatively affected the integrity of the injected blood vessels. Indeed, when CIP1 was pH‐adjusted to a physiological pH, intravenous administration was tolerated at a higher dose of 4 mg/kg.

At a physiological pH, repeated intravenous administration of doses higher than 4 mg/kg also led to local reactions, including swelling, and blackening of the tail. I_2_ is a strong oxidising agent, and high concentrations could lead to local irritation and oxidative injury.^12^

Per‐oral administration of CIP1, at an acidic pH, was tolerable at much higher doses, as compared to intravenous injection. At the highest feasible per‐oral dose, 80 mg/kg, no adverse effects were seen. Interestingly, the pH adjusted CIP1 to a physiological pH reduced the tolerability with a maximum tolerable dose of 26.7 mg/kg. Systemic reactions were observed, including weight loss, piloerection and pre‐term death. The reason for this is unclear, but it is possible that when administering the pH adjusted CIP1 per‐orally, the compound will immediately be exposed to an acidic environment, and the pH adjustment performed ex vivo will be reversed. As a result, the toxic effects seen are unlikely due to the pH of the compound per se, but possibly due to molecular changes of Iodine upon pH adjustment.

### 4.2 Cancer models

No effect on tumour growth was evident for CIP1 in the CT26 model. However, the inability to inhibit CT26 tumour growth is not equivalent to an inability to inhibit cancer growth per se and hence other models were explored. Indeed, intravenous CIP1, at both physiological and acidic pH, and per‐oral CIP1 at physiological pH significantly blunted tumour growth in the xenograft breast cancer model. The most pronounced effect was evident following administration of intravenous CIP1 at an acidic pH. Subsequently, we could also show that all test items had a significant effect on survival in the orthotopic, syngeneic lung metastasis model where the most prominent effect was seen in animals that were subjected to administration of CIP1 at a physiological pH.

Given the challenges with pH adjustment, stability, the intravenous administration route and side effects mainly related to administration and low pH, a buffered colloidal particle system for per oral administration, CIP3, was developed successfully with stability exceeding CIP1. CIP3 was compared to CIP1 in the lung metastasis model, and we could confirm a similar effect on median survival. Post‐mortem evaluation of tumour burden further demonstrated a clear effect of both CIP1 and CIP3, delivered per‐orally leading us to conclude equivalence of CIP3 with superior commercial advantages.

Conventional chemotherapy is linked to many well described severe side effects such as acute and chronic toxicity.^13^, ^14^ Most organs of the body are affected including vital ones such as the heart, lungs and brain. Moreover, chronic effects of chemotherapy can entail drug resistance, carcinogenicity and infertility. Hence, it is interesting to note that the effect of per oral administration of CIP3 was equivalent to that seen following administration of cisplatin but with absence of toxicity.

Interestingly, ancient traditional medicine systems have long used iodine‐rich seaweeds as a cancer treatment and iodine supplementation has been linked to positive reductions in breast and prostate cancer epidemiology.^15^ Despite such promising data, no major clinical translation into a pharmaceutical product has been undertaken. However, a recent pilot study has analysed the adjuvant effect of a water solution of iodine together with chemotherapeutic treatment against breast cancer. The preliminary results indicate that the supplementation of iodine, notably iodine ions, improves the effectiveness of the treatment, decreasing side effects and increasing disease‐free survival specially in advanced conditions (stage III). They were also able to show that iodine supplementation induces tumour re‐differentiation and the reactivation of antitumour immune responses.^16^

It is understandable that without an effective and commercially available pharmacological product aligned with standard operation procedure requirements the impact on standard treatment guidelines in cancer care is scant.

Taken all together, our CIP formulations hold promise to improve cancer treatment based on: A. The NP drug delivery design; B. High concentration of I_2_; C. Tolerability; D. Route of administration; E. Efficacy and; F. Shelf life stability compliant with commercial product requirements.

Finally, preliminary findings from our recent pilot study, which will be reported separately, show that iodine accumulates to a higher degree in tumour‐bearing tissues compared to other tissue, for example muscle tissue, specifically focusing on MDA‐MB‐231 and LL/2 tumours (data not shown).

#### 4.2.1 Methodological considerations

There are several limitations of this study. Evaluation of health status and survival, as well as terminal tumour burden, is not an optimal way to determine efficacy in metastatic models. Indeed, tumour burden could not be evaluated in animals found dead, resulting in a reduction of sample size.

In addition, animals were euthanized at different time‐points, which means that end‐point tumour burden may not be an ideal comparison. This may explain why the number of large tumours in some treatment groups was larger, as compared to the vehicle group. The use of in vivo imaging would allow for continuous quantification of tumour burden on all animals, and this will be considered in future experiments.

Initially all experiments were carried out with CIP1 but due to problems of stability and pH adjustments we added citrate as a buffering agent. This improved the shelf‐life stability considerably of the formulation and omitted the need for pH adjustments. In the follow‐up study, that was performed to validate the findings of CIP1 delivered at physiological pH, we included the new buffered formulation of CIP; CIP3. Here we were able to demonstrate equivalent efficacy. However, in future experiments we will consider including a full tolerability assessment of CIP3 to determine whether higher doses of this formulation can be delivered without inducing toxic effects. In addition, the effect of CIP3 in both colon and breast cancer models may also be repeated.

Moreover, previous research has analysed the effect of I_2_ in human breast cancer cell lines with low (MCF‐7) and high (MDA‐MB231) metastatic potential under both in vitro (cell proliferation and invasion assay) and in vivo (xenografts of athymic nude mice) conditions^2^ and future research should also investigate the effect on cell lines to elucidate specific effects including mechanisms of action pertaining to the NP I_2_ formulations.

Finally, the reported size of the colloidal nanoparticles has been evaluated by the dynamic light scattering (DLS) method using a Malvern Nano Zetasizer. Validation and additional characterisation should be performed in future studies using for example scanning electron microscopy (SEM) or transmission electron microscopy (TEM).

## 5 CONCLUSION

Despite the formulation challenges, we were successful in constructing stable nano particles (NPs) loaded with I_2_, which have convincing commercial applicability. Taking in consideration the methodological constraints of this study we can conclude that administration of the NP I_2_ drug delivery system: 1. Blunted tumour growth in a xenograft breast cancer model; 2. Had a significant effect on survival in the orthotopic, syngeneic lung metastasis model; 3. Showed reduced tumour burden in post‐mortem evaluation and; 4. Was associated with low degrees of side effects. Taken all together, our findings indicate that the NP I_2_ drug delivery system may serve as a novel and effective cancer treatment with low degree of side effects. This is something, which needs further exploration and confirmation in future clinical trials. In addition, future experiments should also address mechanisms of action of our NP I_2_ formulations in vitro and in vivo.

## AUTHOR CONTRIBUTIONS

**Torkel Falkenberg:** Conceptualization (equal); formal analysis (lead); investigation (lead); project administration (equal); supervision (lead); writing – original draft (lead); writing – review and editing (lead). **Olivia Larsson:** Data curation (lead); formal analysis (equal); investigation (equal); methodology (lead); validation (lead); writing – original draft (equal); writing – review and editing (equal). **Bengt Hedin:** Data curation (equal); formal analysis (equal); investigation (equal); methodology (lead); supervision (equal); validation (lead); writing – original draft (equal); writing – review and editing (equal). **Shigeru Shiraki:** Conceptualization (lead); funding acquisition (lead); project administration (equal); resources (lead); supervision (equal); writing – original draft (equal); writing – review and editing (equal). **Takahisa Karita:** Conceptualization (lead); formal analysis (equal); funding acquisition (lead); investigation (lead); project administration (equal); resources (lead); supervision (equal); visualization (equal); writing – original draft (equal); writing – review and editing (equal).

## FUNDING INFORMATION

This work was financed by Shigeru AG, Gerbergasse 48 CH‐4001 Basel, Switzerland.

## CONFLICT OF INTEREST STATEMENT

Colloidal Iodine is being developed by Shigeru AG which financed the research and development performed by Adlego/Scantox and APL without any demands of retribution

## ETHICS STATEMENT

All appropriate ethical approvals were sought and approved.

## Data Availability

Data is available upon request.
